# Efficacy profile of noninvasive vagus nerve stimulation on cortical spreading depression susceptibility and the tissue response in a rat model

**DOI:** 10.1186/s10194-022-01384-1

**Published:** 2022-01-21

**Authors:** Tzu-Ting Liu, Andreia Morais, Tsubasa Takizawa, Inge Mulder, Bruce J. Simon, Shih-Pin Chen, Shuu-Jiun Wang, Cenk Ayata, Jiin-Cherng Yen

**Affiliations:** 1 Institute of Pharmacology, College of Medicine, National Yang Ming Chiao Tung University, 5th floor, Shouren Building, No. 155, Sec. 2, Linong St., Beitou District, 112 Taipei, Taiwan; 2 Neurovascular Research Lab, Department of Radiology, Massachusetts General Hospital, Harvard Medical School, Charlestown, MA USA; 3 National Institute of Translational Neuroscience, Biomedical Science Institute, Federal University of Rio de Janeiro, Rio de Janeiro, RJ Brazil; 4 electroCore, Inc, Basking Ridge, NJ USA; 5 Department of Neurology, Taipei Veterans General Hospital, Taipei, Taiwan; 6 Institute of Clinical Medicine, College of Medicine, National Yang Ming Chiao Tung University, Taipei, Taiwan; 7 Department of Medical Research, Taipei Veterans General Hospital, Taipei, Taiwan; 8 Faculty of Medicine, College of Medicine, National Yang Ming Chiao Tung University, Taipei, Taiwan; 9 Brain Research Center, National Yang Ming Chiao Tung University, Taipei, Taiwan

**Keywords:** Cortical spreading depression, Vagus nerve stimulation, Neuroinflammation, Trigeminal activation

## Abstract

**Background:**

Noninvasive vagus nerve stimulation (nVNS) has recently emerged as a promising therapy for migraine. We previously demonstrated that vagus nerve stimulation inhibits cortical spreading depression (CSD), the electrophysiological event underlying migraine aura and triggering headache; however, the optimal nVNS paradigm has not been defined.

**Methods:**

Various intensities and doses of nVNS were tested to improve efficacy on KCl-evoked CSD frequency and electrical threshold of CSD in a validated rat model. Chronic efficacy was evaluated by daily nVNS delivery for four weeks. We also examined the effects of nVNS on neuroinflammation and trigeminovascular activation by western blot and immunohistochemistry.

**Results:**

nVNS suppressed susceptibility to CSD in an intensity-dependent manner. Two 2-minute nVNS 5 min apart afforded the highest efficacy on electrical CSD threshold and frequency of KCl-evoked CSD. Daily nVNS for four weeks did not further enhance efficacy over a single nVNS 20 min prior to CSD. The optimal nVNS also attenuated CSD-induced upregulation of cortical cyclooxygenase-2, calcitonin gene-related peptide in trigeminal ganglia, and c-Fos expression in trigeminal nucleus caudalis.

**Conclusions:**

Our study provides insight on optimal nVNS parameters to suppress CSD and suggests its benefit on CSD-induced neuroinflammation and trigeminovascular activation in migraine treatment.

**Supplementary Information:**

The online version contains supplementary material available at 10.1186/s10194-022-01384-1.

## Background

Migraine ranks as the second leading cause of disability worldwide, imposing a heavy burden on not only individuals but also socioeconomics. Although our understanding of migraine pathophysiology has advanced remarkably over the past decades, dissatisfaction with treatment options remains a challenge due to low efficacy, adverse effects and risk of medication overuse [1]. For example, only 18-50% of patients receiving triptans, one of the most effective abortive drugs for migraine, had a 2-hour pain-free response [2]. Nonpharmacologic approaches, such as vagus nerve stimulation (VNS), have recently emerged as promising alternatives to existing standard treatments [3].

VNS is approved by the U.S. Food and Drug Administration (FDA) for intractable epilepsy and depression. Case reports and small series of patients receiving invasive VNS (iVNS) for intractable epilepsy [4] or depression [5] reported a serendipitous reduction in their concomitant migraine attacks, paving the way for testing VNS in migraine. Noninvasive VNS (nVNS) rendered VNS feasible for a broader population of headache sufferers, exhibiting abortive and prophylactic effects on episodic or chronic migraine [6–8]. Recently, a double-blind, randomized sham-controlled trial has established the efficacy of acute nVNS therapy in episodic migraine comparable to standard treatments, with the added benefit of being well-tolerated [3]. Although another trial showed that preventive nVNS treatment in episodic migraine was not superior to sham stimulation in enrolled patients who received≥1 treatment in the double-blind period, a post hoc analysis showed that nVNS significantly reduced migraine days, headache days, and acute medication days in the population of patients who were ≥ 67% adherent [9]. FDA has approved nVNS for acute treatment and prevention of migraine in adults and adolescents, as well as cluster headache in adults. Vagus nerve contains A-, B- and C-fibers. Activation of different fiber groups require different stimulation parameters, which may influence VNS efficacy [10–12]. However, the diversity of VNS paradigms used in different studies, such as variable intensity, duration, repetition intervals [6, 7, 13, 14], highlighted the need to examine VNS parameters towards optimization.

We have previously shown that VNS acutely inhibits susceptibility to cortical spreading depression (CSD) [15], a neuronal and glial depolarization wave propagating across the cerebral gray matter, as one mechanism that might explain its efficacy in migraine. CSD is generally accepted as the pathophysiological event underlying migraine aura and triggering headache [16, 17]. A clinical trial showed that reduction of migraine days with nVNS was higher in patients with aura than in those without aura [9]. Using a validated experimental CSD platform for drug screening and efficacy optimization of migraine therapy [18, 19], we have shown that nVNS is comparable to iVNS in increasing CSD threshold and suppressing CSD frequency [15] via central mechanisms [20]. Here, we tested variations to the nVNS therapeutic paradigm in a rat model towards better understanding its efficacy profile on CSD. In addition, we examined nVNS efficacy on CSD-induced neuroinflammation and trigeminovascular activation, possible pathophysiological substate of migraine pain.

## Material & methods

### Ethics

All experimental procedures were carried out according to Guide for the Care and Use of Laboratory Animals (National Institutes of Health Publication) and approved by Institutional Animal Care and Use Committee of National Yang-Ming University, Taiwan, or Massachusetts General Hospital Subcommittee on Research Animal Care.

### Animals

A total of 113 adult male Sprague-Dawley rats (220-450 g; obtained from BioLasco, Taiwan; Harlan Laboratories, Indianapolis, IN, or Charles River Laboratories, Wilmington, MA, USA) were used in the study. Rats were housed in laboratory animal room (thermostatic control at 22±1 °C, 40–70% humidity, and 12-hour light/dark cycle) and allowed to access to standard rodent diet and water *ad libitum*.

### General surgical procedure

Rats were anesthetized by pentobarbital (50 mg/kg for induction by intraperitoneal injection, 15-20 mg/kg/h for maintenance by intravenous infusion) or isoflurane (4% for induction, 1.5% for maintenance, in 70% N_2_O and 30% O_2_) [15]. Rectal temperature was maintained at 36.9-37.1 °C throughout the experiments by thermostatically controlled heating pad. Rats then underwent tracheostomy and trachea intubation for mechanical ventilation (SAR-830; CWE, Ardmore, PA, USA, in 70% N_2_ and 30% O_2_). The femoral artery and vein were catheterized with polyethylene tubes (PE-50) for arterial pressure recording or arterial blood gas analysis, and continuous intravenous anesthesia. Mean arterial pressure (MAP, mmHg) and heart rate (HR, beat/min) were monitored via a transducer (T844, ADInstruments, Castle Hill, Australia). Arterial blood was collected for detecting pH, pO_2_, and pCO_2_ by a portable blood gas analyzer (i-STAT, Abbott Point of Care Inc, Princeton, NJ USA). Physiological parameters remained within normal range (Table 1).

**Table 1:** Systemic physiology during CSD recording in different nVNS paradigms

	n	pH	pCO_2_ (mmHg)	pO_2_ (mmHg)	BP (mmHg)
Amplitude-response					
Sham stimulation	6	7.41$\pm$0.01	36$\pm$2	100$\pm 3$	94$\pm 3$
Low intensity nVNS	6	7.38$\pm$0.01	41$\pm$1	95$\pm$8	92$\pm$3
Medium intensity nVNS	6	7.38$\pm$0.01	39$\pm$1	102$\pm$6	95$\pm$6
High intensity nVNS	6	7.41$\pm$0.01	36$\pm$2	104$\pm$2	90$\pm$3
Dose-response					
Sham stimulation	8	7.44$\pm$0.01	37$\pm$1	156$\pm$5	98±4
2$\times$2-min nVNS	8	7.44$\pm$0.01	38$\pm$1	147$\pm$5	99$\pm$3
3$\times$2-min nVNS	9	7.45$\pm$0.01	36$\pm$1	152$\pm$3	95$\pm$3
1$\times$6-min nVNS	8	7.44$\pm$0.01	38$\pm$1	150$\pm$5	96$\pm$3
Chronic treatment					
Daily nFNS	6	7.42$\pm$0.01	39$\pm$1	134$\pm$5	107$\pm$4
Daily nVNS	8	7.43$\pm$0.01	35$\pm$1*	143$\pm$4	94$\pm$6

*P=0.023 vs. nFNS (Mann-Whitney U test)

Data are mean$\pm$SEM

### Noninvasive vagus nerve stimulation paradigms

A custom-made gammaCore noninvasive VNS device (a 5-kHz sine wave for 1 millisecond repeating at a rate of 25 Hz, electroCore LLC, Basking Ridge, NJ, USA) was employed to deliver the unilateral stimulation by placing 2 disk electrodes (6 mm in diameter, 5 mm separation) on the shaved and intact skin covering the right vagal nerve (5‒8 mm lateral to midline from larynx) with appropriate conductive gel to assure a close contact. After VNS, rats were placed in stereotaxic frame (Stoelting, Wood Dale, IL, USA) for craniotomy and subsequent CSD recording. Three variations of VNS parameters were tested: amplitude-response (single VNS at low, medium or high intensity, corresponding to output voltages of 1 V, 11.4 V, and 24.4 V, respectively, for 2 min), duration-response (two or three consecutive 2-minute VNS at 11.4 V, 5 min apart, or single 6-minute VNS at 11.4 V), and chronic daily VNS (two consecutive 2-minute VNS at 11.4 V, 5 min apart) for 4 weeks [18]. Noninvasive femoral nerve stimulation (nFNS) in the anterior thigh area overlying the quadriceps femoris muscle was used as control.

### CSD susceptibility

Three burr holes were drilled under saline cooling (from bregma) over occipital (4.5 mm posterior, 2 mm lateral, 2 mm diameter for stimulation), parietal (1.5 mm posterior, 2 mm lateral, 1 mm in diameter for recording site 1) and frontal cortex (2 mm anterior, 2 mm lateral, 1 mm in diameter for recording site 2). The dura was kept intact for the recording sites but removed at the stimulation site. Electrocorticogram and direct coupled (DC) potential was recorded between glass micropipettes 0.3~0.5 mm below the surface of dura and a ground electrode under the cervical skin, using Ag/AgCl electrodes inserted into the glass micropipettes (filled with 0.9% NaCl) and a DC amplifier (EX1 differential amplifiers; Dagan Corporation, Minneapolis, MN, USA). The signal was continuously digitized and stored for offline analyses (PowerLab; ADInstruments, Colorado Springs, CO, USA). Characteristic DC potential shifts greater than 5 mV were taken as CSD events. To determine the electrical CSD threshold, single-square pulses of increasing duration and intensity (50-4000 µC) were applied to the pia mater using a bipolar electrode (400 μm tip diameter, 1 mm tip separation; Frederick Haer Company, Bowdoin, ME, USA) every 5 min until a CSD was triggered. VNS effect on electrical threshold of CSD was presented as percentage of difference between groups, i.e., [1-(electrical threshold in sham stimulation group/electrical threshold in VNS group)]⋅100%. To determine KCl-induced CSD frequency, multiple recurrent CSDs were induced by continuous topical application of a KCl-soaked cotton ball (1 M in 0.9% NaCl, 1.5-2 mm in diameter) replaced every 15 min until the end of the recording. The concentration of KCl used in previous studies ranges from 0.5 M to 5 M depending on experimental design, but low concentration of KCl (<1 M) may remain a concern for insufficiency of CSD induction in rats [21, 22] and high concentration up to 5 M causes cortical lesions [23]. Therefore, we chose 1 M KCl, a commonly used concentration, to induce CSD. CSD amplitude and duration were also quantified.

### Western blot

**Fig. 1:**
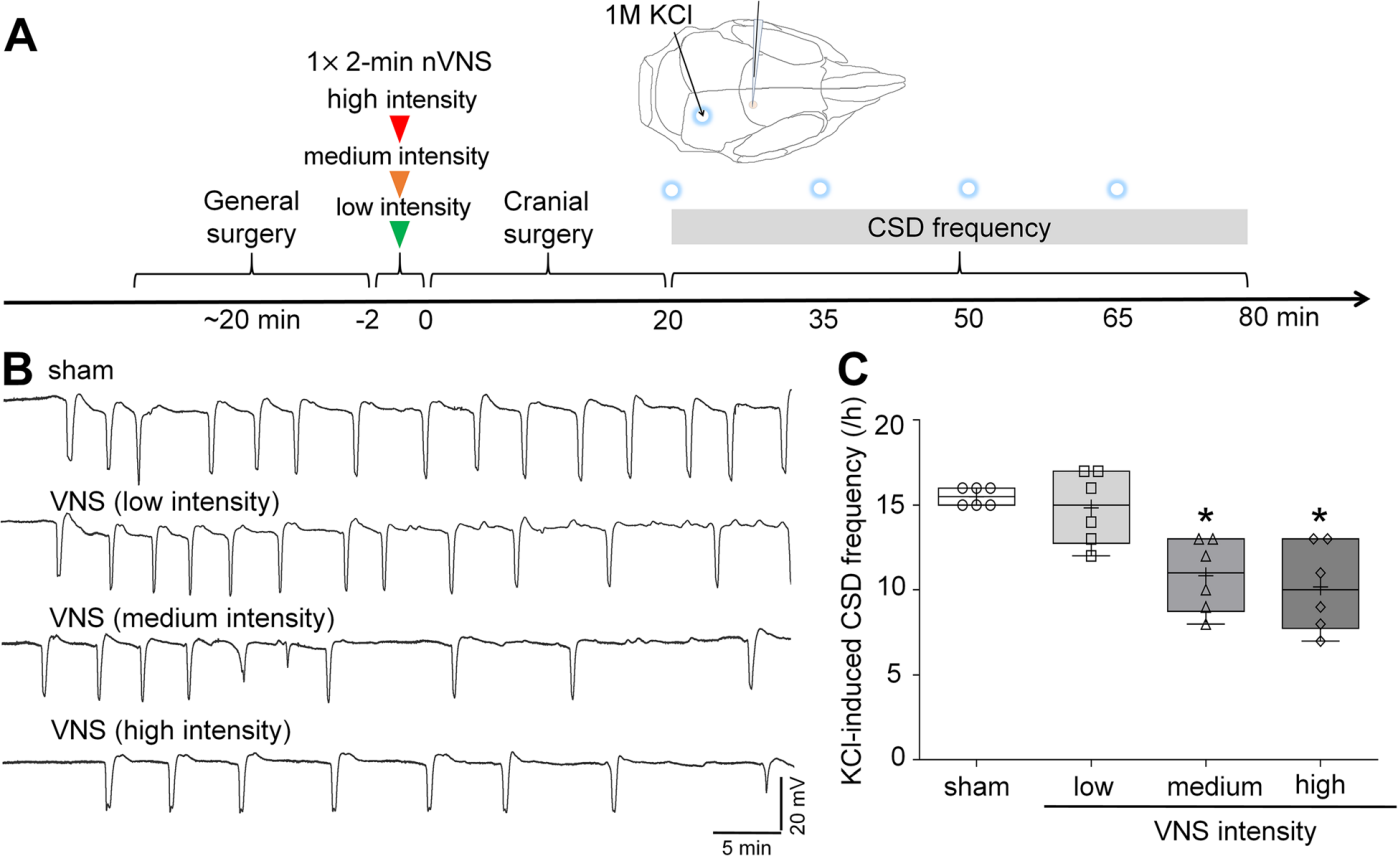
nVNS intensity-dependently suppresses KCl-evoked CSD frequency. **A** Schematic diagram of amplitude-response paradigm of acute nVNS. **B** Representative intra-cortical microelectrode recordings show the effect of sham stimulation or noninvasive transcutaneous VNS at low (1 V for 2 min), medium (11.4 V for 2 min), high intensity (24.4 V for 2 min) on KCl (1 M)-evoked CSD frequency in rats. **C** Whisker–box plots show that medium and high intensity VNS but not low voltage VNS or sham control inhibit KCl-evoked CSD frequency in rats (*n*=6 per group) (whisker: full range; line: median; cross: mean; ^*^P=0.0349 for medium intensity, ^*^P= 0.0149 for high intensity, compared to sham group, Kruskal-Wallis test followed by *post hoc* Dunn’s multiple comparisons test)

After CSD induction (from bregma, 5 mm posterior, 2 mm lateral for KCl site; 2 mm anterior, 2 mm lateral for recording site) for 1 h (a separate group of rats) or 2 h (samples from rats shown in Fig. 1), the cerebral cortex (bregma 0 mm to -2 mm) was harvested at 2 h. The anesthetized rats were perfused transcardially with ice-cold saline and brain was removed immediately. Cortex between KCl stimulation and posterior recording site was collected and homogenized (0.5 mm zirconium oxide bead, Next Advance Inc, Troy, NY, USA) in lysis buffer (GBioscience#786-181, St. Louis, MO, USA) containing protease inhibitor (Roche#04693132001, Basel, Switzerland). The homogenate was kept on ice for 30 min for lysis and then centrifuged at 4℃, 10,000 xg for 15 min. The supernatant, the total cell lysate, was collected and the protein concentration was determined using Bradford protein assay. Cell lysate was diluted in 4× Laemmli buffer (250 mM Tris–HCl pH 6.8, 10% (w/v) SDS, 5% (v/v) 2-mercaptoethanol, 25% (v/v) glycerol, 0.1% (w/v) bromophenol blue) and denatured at 110℃ for 10 min. Each protein sample (40 µg per lane) was loaded and separated by 10% sodium dodecyl sulfate polyacrylamide gel electrophoresis (SDS-PAGE). Protein was then transferred onto polyvinylidene difluoride membrane by wet electroblotting, conducted in ice-cold transfer buffer at 150 V and 350 mA for 1.5 h at 4℃ and followed by blocking in 5% fat-free milk at room temperature for 1 h. After blocking, membranes were adequately trimmed and incubated in primary antibodies: rabbit anti-cyclooxygenase-2 (COX-2; 1:1000; Abcam#ab15191, Cambridge, MA, USA) or mouse anti-β-actin (1:5000; Sigma-Aldrich#A5441, St. Louis, MO, USA) at 4℃ for 12-16 h. The membrane was followed by rinse and incubation with horseradish peroxidase (HRP)-conjugated donkey anti-rabbit IgG (1:2500; GE Healthcare#NA934, Chicago, Illinois, USA) or sheep anti-mouse IgG (1:2500; GE Healthcare#NA931) in 3% fat-free milk at room temperature for 1 h. Finally, the band signal was developed using Immobilon Western Chemiluminescent HRP Substrate (Merck Millipore#WBKLS0500, Burlington, MA, USA), captured by Luminescence/Fluorescence Imaging System (GE Amersham Imager 680), and quantified by Image J analysis software. Intensity of COX-2 was normalized against the intensity of corresponding β-actin, the loading control, on the same membrane.

### Immunochemistry and immunofluorescence staining

A separate group of rats were used for histology. After CSD induction for 1 h (from bregma, 5 mm posterior, 2 mm lateral for KCl site; 2 mm anterior, 2 mm lateral for recording site), KCl was washed out and the trigeminal ganglia (TG) and trigeminal nucleus caudalis (TNC) were harvested at 2 h. The anesthetized rats were transcardially perfused with 37℃ normal saline followed by ice-cold 4% paraformaldehyde. The brain was rapidly removed, fixed in 4% paraformaldehyde at 4℃ for 24 h, and serial dehydrated in 15% sucrose solution for 24 h and then in 30% sucrose solution at 4℃ until brain sank. TG sections (20 μm) were mounted onto slides, blocked in 3% normal goat serum (diluted in PBS buffer containing 0.375% gelatin and 0.2% Triton-X-100) at room temperature for 30 min, and subsequently incubated with primary antibodies: rabbit anti-calcitonin gene-related peptide (CGRP; 1:100; Abcam#ab47027) and mouse anti-NeuN (1:100; Merck Millipore#MAB377) at 4 ºC for 16-24 h. To observe cells expressing cyclooxygenase-2 (COX-2), an inflammatory marker, in cerebral cortex, coronal cerebral sections (20 μm, bregma +1 mm to -1 mm) were blocked with 3% normal donkey serum and incubated with primary antibody: rabbit anti-COX-2 (1:200; Abcam#ab15191), which were co-incubated with mouse anti-NeuN (1:100), mouse anti-glial fibrillary acidic protein (GFAP; an astrocyte marker; 1:150; Sigma-Aldrich#G3893), or goat anti-allograft inflammatory factor 1 (Iba1; a microglia marker; 1:150, Abcam#ab5076) using free-floating staining method. After primary antibody incubation, TG sections were rinsed with PBS and incubated with Alexa Flour 488-conjugated goat anti-mouse IgG (1:100; Invitrogen#A11001, Waltham, MA, USA) and Alexa Flour 594-conjugated goat anti-rabbit IgG (1:100; Invitrogen#A11012). Cortical sections were incubated with Alexa Flour 488-conjugated donkey anti-mouse (1:200; Invitrogen#A21202) or donkey anti-goat IgG (1:200; Invitrogen#A11055) and Alexa Flour 594-conjugated donkey anti-rabbit IgG (1:200; Invitrogen#A21206) at room temperature for 2 h prior to mounting the coverslips with antifade mounting medium (Vector Laboratories#H-1500; Burlingame, CA, USA), and imaged using laser confocal microscope (Olympus FV1000). The number of neuronal CGRP-positive cells in the center of three TG sections (section interval :100 μm) were analyzed in a square area (200 × 200 µm^2^, field of view at 600X magnification) using Image J software for fluorescent intensity threshold setting. NeuN staining was employed to identify neuronal nuclei and to confirm that the field of view contained an equal number of neurons. Background signals were acquired from nonneuronal area (identified by NeuN staining) and subtracted from the regions of interest. For c-Fos expression, a marker of neuron activation, in TNC, coronal section (20 μm) were collected and blocked as mentioned above. The free-floating sections were incubated with rabbit anti-c-Fos antibody (1:500, Cell Signaling Technology #2250, Danvers, MA, USA) for 16-24 h at 4℃. Afterward, the sections were incubated with biotinylated goat anti-rabbit IgG at room temperature for 1 h and then transferred to PBS buffer containing avidin-biotin complex at room temperature for 0.5 h (Vector Laboratories#PK-6102). Finally, black immunoreactivity in cells expressing c-Fos was visualized using a nickel-enhanced diaminobenzidine substrate kit (Vector Laboratories#PK-4100). After staining, the sections were mounted onto slides, serially dehydrated with ethanol, cleared with xylene, and applied to mounting medium (Sigma-Aldrich#06522) with coverslips. The images were visualized under optical microscope (Olympus BX63, field of view at 100X magnification) and analyzed by MShot Image analysis system (MD60). c-Fos-immunoreactivity in lamina I and II of TNC ipsilateral to CSD induction site were analyzed in five random sections from caudal medulla (bregma -15 mm) to spinal cord C1 level. Three independent staining were carried out, averaged, and presented as the number of cells or the percentage of neuronal cells. Negative controls were carried out with the same staining procedures but with omission of primary antibodies.

### Statistical analysis

Rats were randomly assigned to each group. No animal was excluded for poor physiology. Sample sizes were calculated using GPower (version 3.1) to detect an effect size of 50% and achieve 80% power (α = 0.05). Data were analyzed by an independent researcher blinded to the study arms and presented as whisker-box plots (whisker: full range; box: interquartile range; line: median; +: mean) as shown in figures. Shapiro–Wilk test was used for normality test. For parametric variables, unpaired t test with Welch’s correction was used for significance comparison between two groups without assumption of equal variances and one-way analysis of variance (ANOVA) followed by *post hoc* Bonferroni test were used for multiple comparison. Non-normally distributed variables were analyzed by Mann-Whitney U test or Kruskal–Wallis test followed by *post hoc* Dunn’s test. *P*<0.05 was considered statistically significant. Statistical analysis was performed using IBM SPSS Statistics (version 24, IBM, Armonk, NY, USA) or GraphPad Prism (version 9, Graphpad Software Inc, San Diego, CA, USA).

## Results

### Noninvasive nVNS had a quasi-dose effect on CSD

**Fig. 2:**
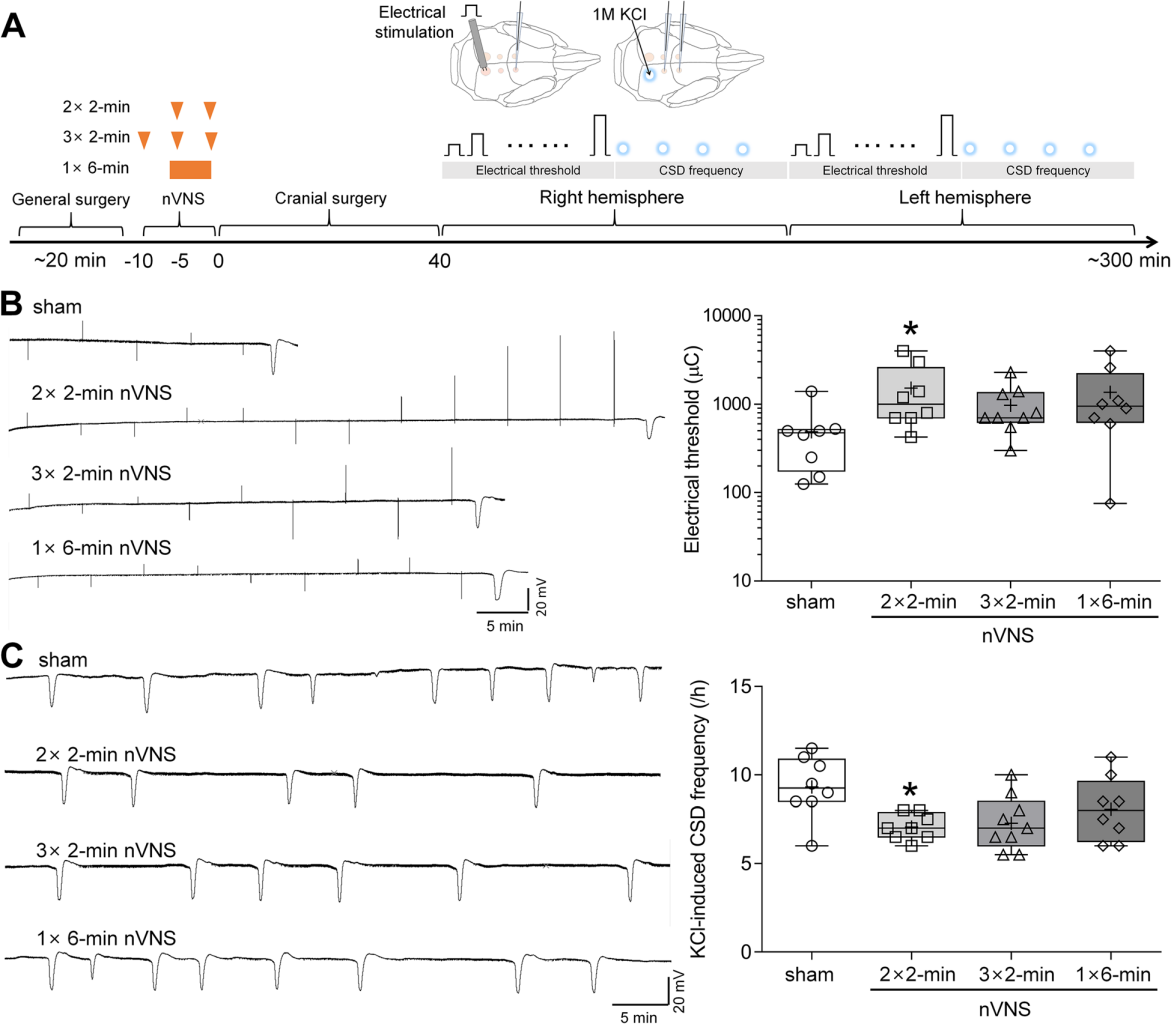
Two 2-minute nVNS affords the most efficacious suppression of CSD. **A** Schematic diagram of dose-response paradigm of acute nVNS. A triangle indicates a 2-minute stimulation at medium intensity. **B-C** Representative intra-cortical microelectrode recordings and whisker–box plots show the effect of sham stimulation or noninvasive transcutaneous VNS at dose of 2 × 2-minute, 3 × 2-minute, and 1 × 6-min (11.4 V) on electrical threshold (**B**) and KCl (1 M)-evoked CSD frequency (**C**) in rats. Whisker–box plots show that 2 × 2-minute nVNS but not 3 × 2-minute, 1 × 6-min or sham stimulation significantly elevate electrical threshold and inhibit KCl-evoked CSD frequency in rats (whisker: full range; line: median; cross: mean; *n*=9 for 3 × 2-minute paradigm and *n*=8 for other groups; data are the mean of two hemispheres; ^*^P=0.0261 for electrical threshold, ^*^P=0.01 for CSD frequency, compared to sham group, Kruskal-Wallis test followed by *post hoc* Dunn’s multiple comparisons test). The lower CSD frequency stems from different anesthesia used in different experimental paradigms (barbiturate for amplitude-response; isoflurane+N_2_O for dose-response and chronic treatment)

VNS suppressed KCl-induced CSD frequency by 30% at medium (11.4 V) and 34.4% at high (24.4 V); low intensity VNS (1 V) was ineffective, suggesting a threshold effect (Fig. 1). Since medium intensity had efficacy comparable to high intensity VNS, we used medium intensity VNS for the following experiments. We next tested three VNS delivery paradigms: two 2-minute VNS 5 min apart (2 × 2-minute), three 2-minute VNS 5 min apart (3 × 2-minute), and one 6-minute VNS (1 × 6- minute) on CSD susceptibility (Fig. 2 A). Although all three paradigms suppressed CSD susceptibility, 2⋅2-minute paradigm appeared marginally more efficacious than the others (68% higher electrical CSD threshold, Figs. 2B and 24% lower KCl-induced CSD frequency, Fig. 2C). Based on these results, 2 × 2-minute paradigm was used for subsequent experiments.

### Chronic daily nVNS was not superior to single nVNS on CSD

**Fig. 3:**
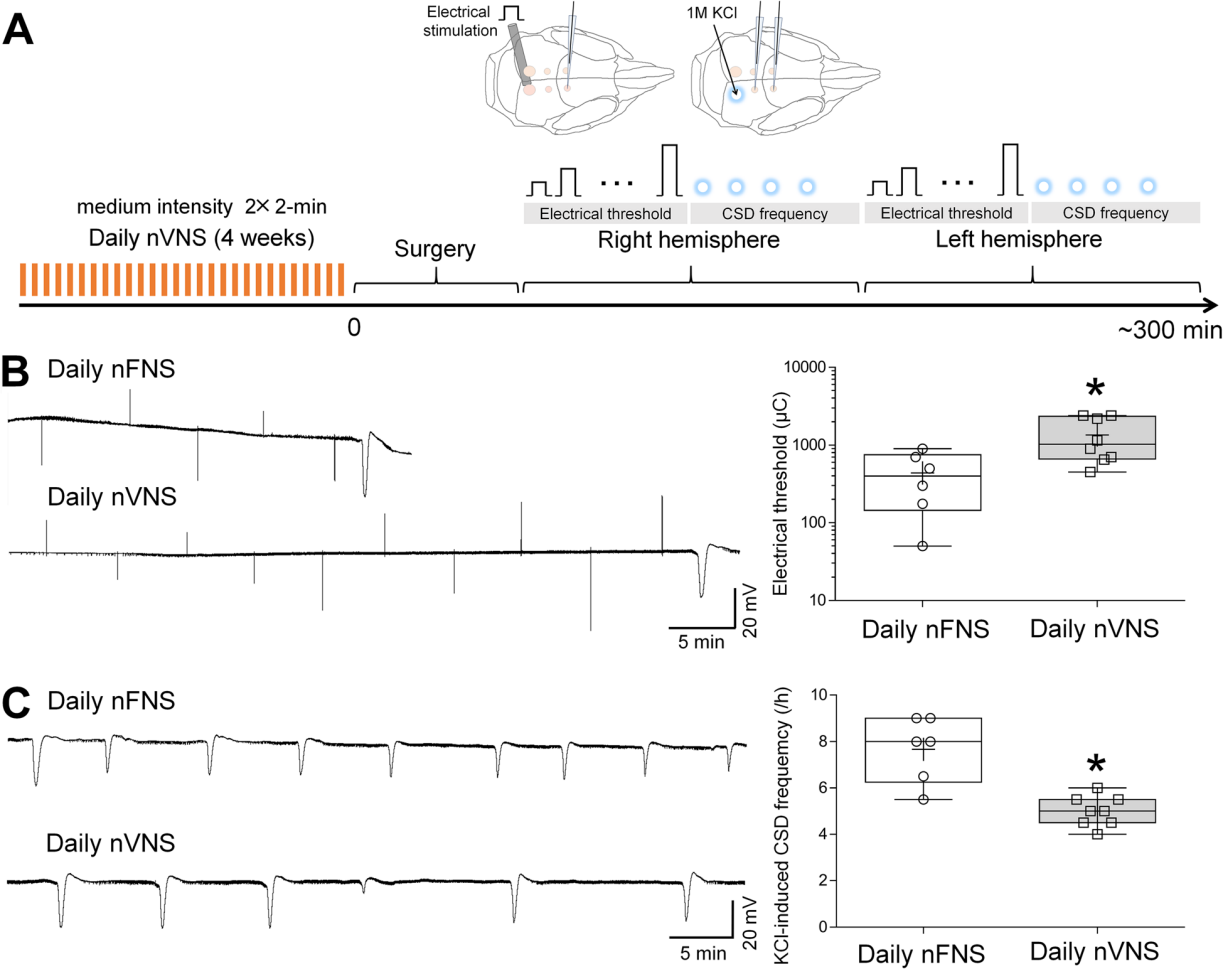
Chronic daily nVNS exerts comparable efficacy to acute treatment. **A** Schematic diagram of chronic daily noninvasive transcutaneous VNS paradigm. A Bar indicates 2 × 2-minute stimulation (**B-C**) Representative intra-cortical microelectrode recordings and whisker–box plots show the effects of noninvasive femoral nerve stimulation (nFNS) or noninvasive transcutaneous VNS (2 × 2-minute, 11.4 V) on electrical threshold (**B**) and KCl (1 M)-evoked CSD frequency (**C**) in rats. Whisker–box plots show that chronic daily nVNS significantly elevates electrical threshold and inhibits KCl-evoked CSD frequency in rats (whisker: full range; line: median; cross: mean; ^*^P=0.0184 for electrical threshold, ^*^P=0.0039 for CSD frequency, compared to daily nFNS group, *n*=6 for nFNS and *n*=8 for nVNS, unpaired-t-test with Welch’s correction), which is comparable to acute treatment (Fig. 2)

Because longer treatment with prophylactic drugs augments CSD suppression [18], we also tested chronic daily VNS for 4 weeks on CSD susceptibility (Fig. 3A). Chronic daily VNS elevated the electrical threshold by 68% (Fig. 3B) and decreased CSD frequency by 35% (Fig. 3 C) compared with FNS. These data suggested that chronic daily VNS for 4 weeks did not significantly augment efficacy over a single VNS tested acutely on CSD. VNS did not affect SD amplitude or duration in any of the cohorts (Table 2).

**Table 2:** Amplitude and duration of CSD in different nVNS paradigms

	n	CSD amplitude (mV)	CSD duration (s)
Amplitude-response			
Sham stimulation	6	21$\pm$1.5	19$\pm$1.7
Low intensity nVNS	6	25$\pm$0.8	15$\pm$0.6
Medium intensity nVNS	6	21$\pm$2.5	18$\pm$1.9
High intensity nVNS	6	19$\pm$2.4	21$\pm$2.7
Dose-response			
Sham stimulation	8	20$\pm$0.6	23$\pm$3.8
2$\times$2-min nVNS	8	20$\pm$1.3	21$\pm$0.7
3$\times$2-min nVNS	9	21$\pm$1.1	21$\pm$1.3
1$\times$6-min nVNS	8	20$\pm$0.8	21$\pm$1.0
Chronic treatment			
Daily nFNS	6	20$\pm$0.4	20$\pm$0.4
Daily nVNS	8	20$\pm$0.7	21$\pm$0.5

Data are mean$\pm$SEM. *P*>0.05 in all comparison (student’s t-test)

### nVNS inhibited cortical neuroinflammation and trigeminovascular activation

**Fig. 4:**
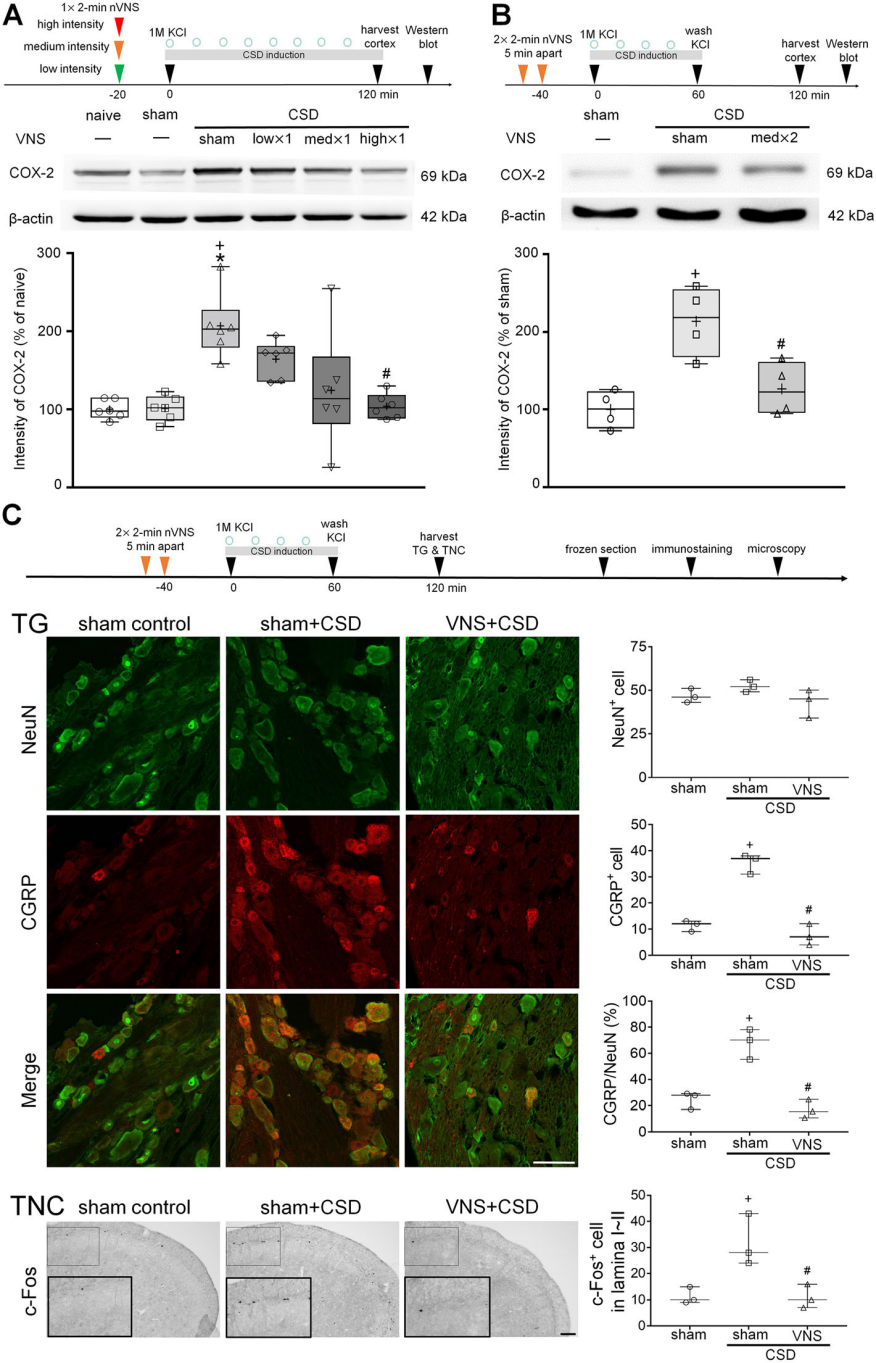
nVNS attenuates CSD-triggered neuroinflammation and trigeminovascular system activation. **A** CSD-induced ipsilateral cortical cyclooxygenase-2 (COX-2) expression in naïve, sham control, and rats receiving CSD induction for 2 h following sham VNS, single 2-minute nVNS at low (1 V), medium (11.4 V), or high intensity (24.4 V). CSD increased ipsilateral cortical COX-2 expression (^*^P=0.0083 versus naïve group; ^+^*P*=0.0137 versus sham control), which were intensity-dependently reduced by nVNS (^#^*P*=0.0167 high intensity×1 VNS+CSD versus sham VNS+CSD group, *n*=6) (**B**) Cortical COX-2 levels in rats receiving sham VNS or VNS (11.4 V, 2$\times$2-minute) followed by CSD induction for 2 h. CSD-induced upregulation of COX-2 attenuated by VNS (^+^*P*=0.0042 versus sham control; ^#^*P*=0.0207 versus sham VNS+CSD group, *n*=4). **C** Confocal images of CGRP expression in ipsilateral TG of rats receiving sham VNS or VNS (11.4 V, 2 × 2-minute) followed by CSD induction for 1 h and the TG were harvested at 2 h. Scale bar indicates 50 μm. CSD upregulated CGRP expression in the TG (^+^*P*=0.0004 for CGRP^+^ cell, ^+^*P*=0.0028 for percentage of neuronal CGRP, compared to sham control), which was attenuated by VNS (^#^*P*=0.0002 for CGRP^+^ cell; ^#^*P*=0.0012 for percentage of neuronal CGRP, compared to sham VNS+CSD group, *n*=3). Immunohistochemistry images show c-Fos in lamina I and II of TNC ipsilateral to CSD induction site from rats receiving sham VNS or VNS (11.4 V, 2$\times$2-minute) followed by CSD induction for 1 h and TNC was harvested at 2 h. Inset rectangles show the magnified images. Scale bar indicates 100 μm. CSD increased c-Fos immunoreactivity in the lamina I~II of TNC (^+^*P*=0.0282 versus sham control group), which was attenuated by VNS (^#^*P*=0.0263 versus sham VNS+CSD group, *n*=3). Data are presented as whisker–box plots with all points (whisker: full range; line: median; cross: mean). Kruskal-Wallis test followed by *post hoc* Dunn’s test (**A**) and one-way ANOVA followed by *post hoc* Bonferroni test (**B-D**) were used for statistical analysis

CSD-induced trigeminovascular system activation provides a direct link of CSD to migraine pathophysiology [17, 24], which is possibly mediated by cortical inflammatory response [25, 26]. Therefore, we next examined VNS on the expression of COX-2 in cerebral cortex, CGRP in TG, and c-fos in the TNC. Two hours of repeated CSD induction using topical KCl (1 M) more than doubled COX-2 expression in the ipsilateral cortex compared with sham controls and contralateral cortex, primarily in neurons (Fig. 4A, Additional file 1B-E). However, KCl (1 M)-induced CSD for 1 h following sham stimulation or VNS caused no significant change in COX-2 expression in ipsilateral TG and TNC (Additional file 2). Low, medium or high intensity VNS delivered as a single 2-minute train suppressed CSD-induced COX-2 upregulation in the ipsilateral cortex in an intensity-dependent manner. The 2$\times$2-minute medium-intensity (11.4 V) VNS paradigm also attenuated CSD-induced upregulation of neuronal COX-2 expression in the ipsilateral cortex when measured 1 h after the end of 1-hour repeated CSD induction by continuous topical KCl (Fig. 4B, Additional file 1). CSD increased CGRP expression in the ipsilateral TG and c-Fos expression in the ipsilateral TNC (Fig. 4C). The 2$\times$2-minute medium-intensity VNS paradigm completely blocked the upregulation of both CGRP in the ipsilateral TG and c-Fos in the ipsilateral TNC (Fig. 4C). Taken together, VNS appeared to reduce the intensity of cortical and downstream trigeminal response following CSD.

## Discussion

Here, we demonstrate that VNS suppression of CSD susceptibility is intensity-dependent, and two 2-minute VNS 5 min apart is as efficacious than longer stimulation paradigms including chronic daily VNS for 4 weeks. The same set of VNS paradigms also suppressed CSD-induced upregulation of cortical and trigeminal molecular markers.

We herein identify an efficacious medium intensity of noninvasive VNS paradigm, in line with prior work showing efficacy in stroke, intracranial aneurysms and epilepsy [27, 28]. Noninvasive VNS paradigms in clinical migraine studies vary depending on the purpose. Acute treatment for migraine usually consists of two or three 1.5-2-minute stimulations (3 trials with undefined intensity, 1 trial using 24 V peak voltage and 60mA peak output current) with 3 to 15-min interval achieves 21-34.2% of 2-hour pain-free rate [3, 6, 7, 29], consistent with our optimal 2 × 2-minute paradigm. A study using 1 × 2-minute stimulation (undefined intensity) for acute treatment in adolescent patients with migraine reported a 40.4% of 1-hour pain-free rate [14], in agreement with our results of a single VNS efficacy on CSD (Fig. 1). For fold change in current (µC), electrical CSD threshold elevated by 2 × 2-minute VNS paradigm is slightly higher than that in prior study (3.1 folds versus 2.5 folds). KCl-evoked CSD frequency testing was carried out at 30 min after 2 × 2-minute VNS in a separate group of rats in previous work, and reduced KCl-induced CSD frequency by 40% [15], yet this was 24%, examined after an electrical stimulation-evoked CSD had emerged in the same hemisphere in this study (Fig. 2C). It is possible that VNS efficacy changes over time or the preceding electrical stimulation or the electrically-evoked CSD has increased CSD threshold, which mildly affected the subsequent VNS efficacy on CSD frequency. Longer VNS delivery in our study tended to marginally diminish efficacy on CSD. In another study, shortening the interval between VNS trains also decreased efficacy in cortical plasticity [30], which may have implications for the 1$\times$6-minute stimulation paradigm we also tested. For prophylactic treatment of migraine, daily 3-6 × 2-minute stimulations for 4-12 weeks succeeded in migraine relief [9, 13, 31]. Unlike stronger CSD suppression by prolonged duration of some prophylactic drugs such as topiramate and propranolol [18], here we show that CSD suppression by VNS was not enhanced by chronic daily treatment, suggesting that a single 2 × 2-minute VNS probably achieves the maximum suppressive effect on CSD. Interestingly, in one study on stroke recovery, increasing VNS delivery reduced efficacy [32]. It is possible that longer VNS delivery may recruit other pathways with opposite effects or desensitize VNS responses, such as G protein-coupled receptors mediating VNS-dependent engagement of the noradrenergic and serotonergic systems [33], possible mechanisms for CSD suppression [34]. Collectively, these data suggest that adequate stimulation intensity, duration, and repetition interval are the determinants of an optimal VNS efficacy.

Low to medium intensity VNS may activate A- and B-fibers, and unmyelinated C-fibers have a higher stimulation threshold above 2 mA [35]. We previously showed that iVNS suppresses CSD at low to medium stimulation intensity (30-second trains of 0.5 millisecond, 0.5 mA square pulses at 20 Hz) [15], which, taken together with data herein, suggests that the CSD suppressive effect of VNS is independent of C-fiber activation. This is also consistent with possible mechanisms involved in the anti-epileptic effect of VNS [36]. Vagal afferents primarily relay in the nucleus tractus solitarius (NTS), which in turn projects to locus coeruleus (LC) and dorsal raphe nuclei (DRN) [37, 38]. We have recently demonstrated that VNS inhibits CSD through activation of these nuclei [20]. Acute VNS at medium intensity is sufficient to activate neurons in NTS and LC, as evident in increased neuronal firing and c-Fos immunoreactivity [39, 40]. A recent study using a relatively high intensity VNS also detected rapidly increased c-Fos expression in DRN [41], although other studies suggest a more delayed DRN activation [39, 40]. In addition, elevated glutamate level is a critical mechanism involved in CSD initiation and propagation. In an ischemia model, acute VNS has been reported to inhibit glutamate release [42]. Altogether, these data suggest that VNS-induced rapid activation of subcortical nuclei or cortical glutamate release may contribute to CSD suppression.

We found elevated expression of COX-2 in cortex, CGRP in TG, and c-Fos in the ipsilateral TNC after CSD. These were consistent with previous work showing that CSD induced cortical COX-2 expression [25, 43], activates trigeminal nerve [24], and upregulated CGRP mRNA and protein expression in TG within 2–4 h [43, 44]. CSD induced COX-2 mRNA [25] and protein (Additional file 1B) upregulation only in the ipsilateral but not contralateral cortex; previous studies have also demonstrated that CSD induced trigeminovascular activation on the ipsilateral side [45, 46], so we only compared VNS efficacy on CSD-induced upregulation of COX-2, CGRP, and c-Fos expression ipsilaterally. VNS with mild to moderate stimulation intensity inhibited neuroinflammation in a rat model of Parkinson’s disease [47]; we also found that VNS inhibited CSD-induced neuroinflammation in an intensity-dependent manner, which indicates VNS parameters will affect its efficacy on neuroinflammation. In models such as nitroglycerin infusion or electrical stimulation at TG, COX-2 was significantly upregulated in the dura mater, TG, and TNC [48]; however, our results showed that topical application of KCl (1 M)-induced CSD did not cause significant change in COX-2 expression in the TG and TNC (Additional file 2). Although we cannot exclude the possibility that insufficient time of tissue harvest after CSD induction contributes to this result, the different findings suggest that the CSD-induced inflammation in the trigeminovascular system may be not equivalent to that in nitroglycerin-induced migraine model. The mechanisms underlying long-lasting trigeminal neuronal activation and headache remains poorly understood, but a currently proposed hypothesis is that CSD-induced release of proinflammatory mediators or upregulation of matrix metalloproteinases gives access to meningeal trigeminal afferents, which leads to sustained trigeminovascular activation [49, 50]. We found that VNS partially inhibited CSD but nearly completely inhibited CSD-induced CGRP and c-Fos upregulation, which suggests VNS may have direct effects on trigeminovascular system.

One previous study using a cervical muscle inflammation and pungent odor-induced episodic migraine model showed that 2 × 2-minute with an interval of 2 h VNS (undefined intensity) suppressed upregulation of phosphorylated extracellular signal-regulated kinases (ERK) in TG [51], which is translocated to the nucleus to increase CGRP-specific enhancer activity [52]. In a nitroglycerin-induced allodynic mouse model, nVNS (2-minute) reversed nitroglycerin-induced high level of glutamate in the TNC, a marker for increased trigeminal pain, which may be one of the mechanisms involved in inhibition of c-Fos expression in the TNC by VNS [53]. In an awake formalin-induced trigeminal pain model, iVNS also inhibited c-Fos immunoreactivity in the TNC [54]. In episodic migraine models induced by pungent odor exposure or nitric oxide, 2 × 2-minute with an interval of 5 min nVNS (undefined intensity) inhibited trigeminal activation by enhancing inhibitory descending pain modulation, which involves GABA_A_, 5-HT3, and 5-HT7 receptors activation [55]. Of note, inhibition of CSD-induced CGRP and c-Fos upregulation by VNS may not entirely reflect the effects of VNS on trigeminovascular pathways, since previous studies have reported that VNS could activate several nuclei involved in trigeminal nociceptive transmission, including parabrachial nucleus, hypothalamus, thalamus, and insula [39, 56, 57]. However, how the nuclei triggered by VNS interact and influence VNS efficacy need further studies. Collectively, it is unclear whether the inhibitory effect of VNS on CSD-induced neuroinflammation and trigeminovascular activation reflects reduced CSD burden or the direct inhibition of neuroinflammatory responses, but the near complete inhibition of these markers suggests the latter.

Our study has limitations. First, migraine is more prevalent in females, but only male rats were used in this study. However, clinical VNS paradigms for migraine patients do not differ between genders. Furthermore, except for cardiac activity, no significant correlation between gender and VNS efficacy on neuronal activity or inflammation has been reported in literature [3, 58, 59]. Second, together with our prior work, nVNS delivered at 20-40 min before CSD susceptibility testing exhibits suppressive effect on CSD, suggesting that VNS efficacy begin at 20-40 min. As timeline and results shown in Fig. 2, CSD suppressive effect of VNS is bilateral since no significant difference in electrical threshold and SD frequency was found between two hemispheres, lasting at least for 5 h. Inhibition of CSD-induced trigeminal activation at 2 h by VNS (Fig. 4C) also supports clinical VNS efficacy on 2-hour pain-free rate. However, temporal profile of VNS efficacy needs to be investigated to better understand the accurate onset, when VNS efficacy peaks, and how long the effect lasts. Lastly, our study was carried out under barbiturate (Figs. 1 and 4) or isoflurane (Figs. 2 and 3) anesthesia. Barbiturate does not suppress CSD [60, 61], but isoflurane has been reported to inhibit CSD [60], increase noradrenergic activity [62], and decrease serotonergic activity [63]. Despite the limitations of the direct effect of isoflurane on CSD and its opposite influences on the mechanisms by which VNS inhibits CSD [20], our results show different anesthetic regimens did not affect VNS efficacy on CSD. Consistent with previous study, chronic VNS under isoflurane did not change systemic physiology [27].

## Conclusions

We provide insight on optimal VNS parameters to suppress CSD, depending on adequate combination of intensity and dose. Acute single VNS treatment is efficacious enough for CSD suppression. With the optimal VNS paradigm, our results suggest benefit on CSD-induced neuroinflammation and trigeminovascular activation in migraine treatment.

## Supplementary information

**Additional file 1.** nVNS attenuates CSD-triggered cortical neuronal cyclooxygenase-2 (COX-2) upregulation. **A** Experimental timeline. **B** Western blot gel shows CSD upregulates COX-2 upregulation in the ipsilateral cortex compared with both sides of cortex of sham control and contralateral cortex after KCl (1 M)-induced CSD for 2 h (*P=0.0029, +*P*=0.0147, and #*P*=0.0051 versus ipsilateral cortical COX-2 in CSD group, *n*=3). Data are presented as whisker–box plots with all points (whisker: full range; line: median; cross: mean). One-way ANOVA followed by *post hoc* Bonferroni test was used for statistical analysis. Immunofluorescence staining shows VNS (11.4 V, 2 × 2-minute) attenuated CSD-induced cortical COX-2 upregulation (field of view at cortical layer II to III, 600X magnification), which was primarily located in neuron (**C**) but not in astrocyte (**D**) or microglia (**E**). CSD was induced for 1 h and cortical tissues were harvested at 2 h. Left panel shows nuclear DAPI staining. Middle panels show green fluorescence-labeled neuronal (NeuN), astrocytic (GFAP), microglial (Iba1) markers, and red fluorescence-labeled COX-2 images. Right panel shows the merge images. Scale bar indicates 50 μm.

**Additional file 2.** CSD with or without noninvasive VNS pretreatment did not cause significant change in COX-2 expression in the trigeminal ganglion (TG) and trigeminal nucleus caudalis (TNC). **A** Experimental timeline. Immunohistochemistry images show COX-2 expression in the ipsilateral TG (**B**) and lamina I-II of TNC (**C**) of rats receiving sham stimulation, sham stimulation+CSD induction for 1 h, or VNS (11.4 V, 2 × 2-minute)+CSD induction for 1 h. Ipsilateral TG were harvested at 2 h. CSD with or without VNS did not cause significant change in COX-2 expression in ipsilateral TG and TNC (*P*>0.05, *n*=3). Data are presented as whisker–box plots with all points (whisker: full range; line: median; cross: mean). Kruskal-Wallis test followed by *post hoc* Dunn’s test was used for statistical analysis. Left panel shows nuclear DAPI staining. Middle panel shows green fluorescence-labeled COX-2, and right panel shows merge images. Scale bar indicates 50 μm (**B**) and 20 μm (**C**), respectively.

## Data Availability

Raw data are available upon request via contacting the corresponding author: jcyen@ym.edu.tw; jcyen@nycu.edu.tw.

## Abbreviations

5-HT: 5-hydroxytryptamine
CGRP: calcitonin gene-related peptide
COX-2: cyclooxygenase-2
CSD: cortical spreading depression
DRN: dorsal raphe nuclei
ERK: extracellular signal-regulated kinases
FDA: U.S. Food and Drug Administration
GFAP: glial fibrillary acidic protein
HRP: horseradish peroxidase
Iba1: allograft inflammatory factor 1
iVNS: invasive vagus nerve stimulation
LC: locus coeruleus
NTS: nucleus tractus solitarius
nVNS: noninvasive vagus nerve stimulation
SDS-PAGE: sodium dodecyl sulfate polyacrylamide gel electrophoresis
TG: trigeminal ganglion
TNC: trigeminal nucleus caudalis
