# Supplementary figures and images for: Efficacy profile of noninvasive vagus nerve stimulation on cortical spreading depression susceptibility and the tissue response in a rat model
Source: J Headache Pain. 2022 Jan 21;23(1):12. doi: 10.1186/s10194-022-01384-1 (PMC8903561; doi:10.1186/s10194-022-01384-1)

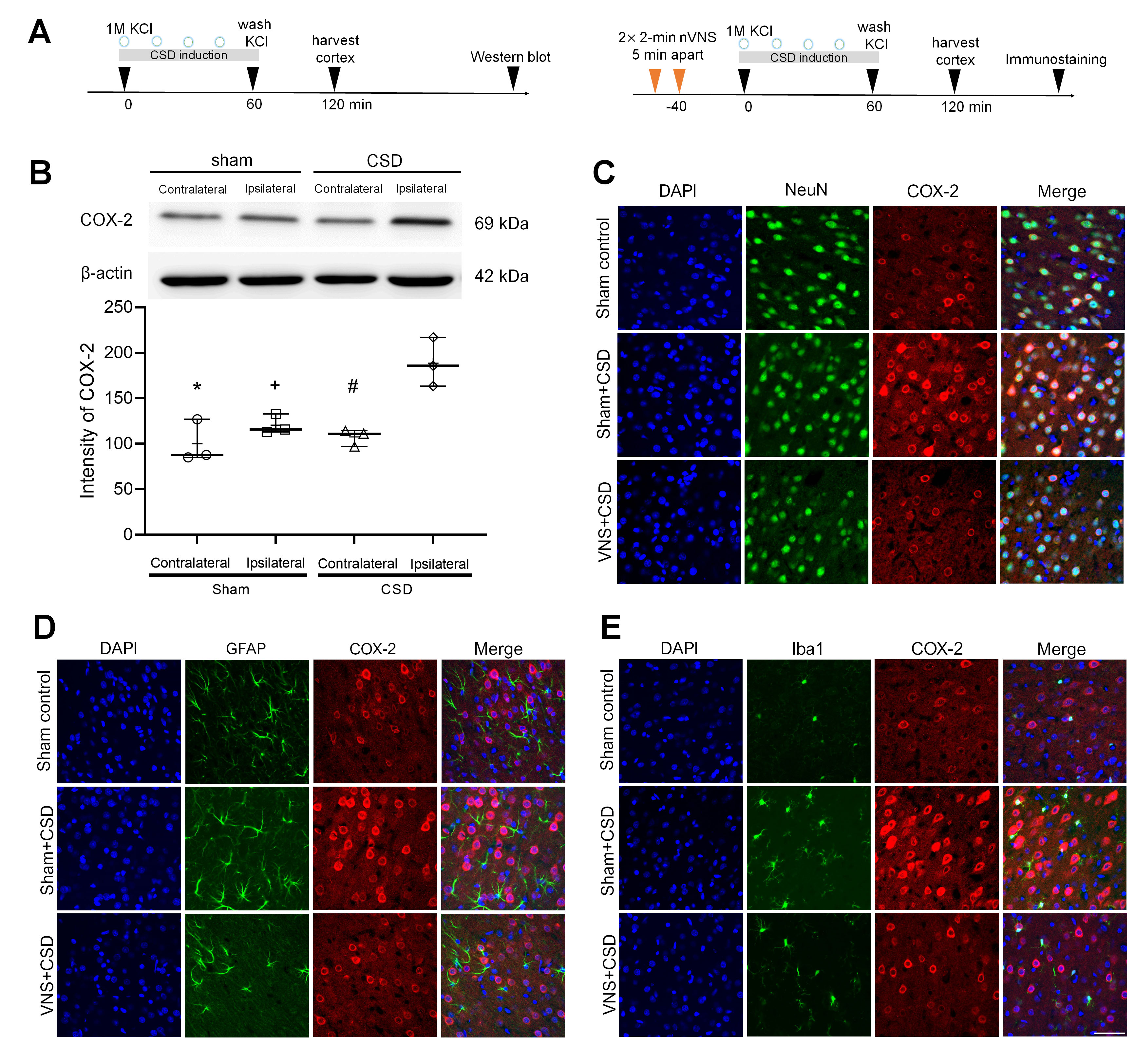

Supplement: Supplementary file 1 — Additional file 1. nVNS attenuates CSD-triggered cortical neuronal cyclooxygenase-2 (COX-2) upregulation. A Experimental timeline. B Western blot gel shows CSD upregulates COX-2 upregulation in the ipsilateral cortex compared with both sides of cortex of sham control and contralateral cortex after KCl (1 M)-induced CSD for 2 h (*P=0.0029, +P=0.0147, and #P=0.0051 versus ipsilateral cortical COX-2 in CSD group, n=3). Data are presented as whisker–box plots with all points (whisker: full range; line: median; cross: mean). One-way ANOVA followed by post hoc Bonferroni test was used for statistical analysis. Immunofluorescence staining shows VNS (11.4 V, 2 × 2-minute) attenuated CSD-induced cortical COX-2 upregulation (field of view at cortical layer II to III, 600X magnification), which was primarily located in neuron (C) but not in astrocyte (D) or microglia (E). CSD was induced for 1 h and cortical tissues were harvested at 2 h. Left panel shows nuclear DAPI staining. Middle panels show green fluorescence-labeled neuronal (NeuN), astrocytic (GFAP), microglial (Iba1) markers, and red fluorescence-labeled COX-2 images. Right panel shows the merge images. Scale bar indicates 50 μm. [file 10194_2022_1384_MOESM1_ESM.jpg]

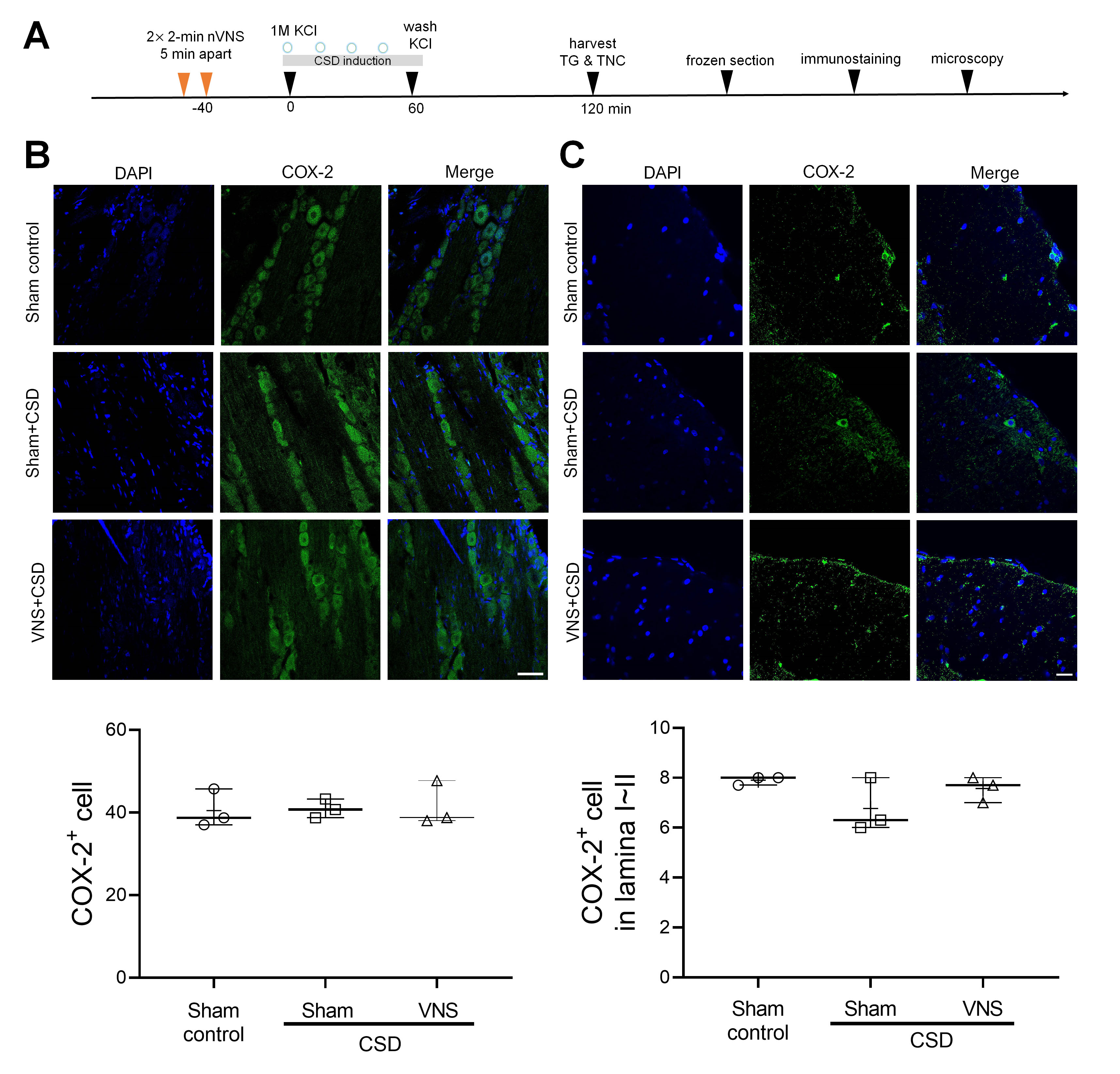

Supplement: Supplementary file 2 — Additional file 2. CSD with or without noninvasive VNS pretreatment did not cause significant change in COX-2 expression in the trigeminal ganglion (TG) and trigeminal nucleus caudalis (TNC). A Experimental timeline. Immunohistochemistry images show COX-2 expression in the ipsilateral TG (B) and lamina I-II of TNC (C) of rats receiving sham stimulation, sham stimulation+CSD induction for 1 h, or VNS (11.4 V, 2 × 2-minute)+CSD induction for 1 h. Ipsilateral TG were harvested at 2 h. CSD with or without VNS did not cause significant change in COX-2 expression in ipsilateral TG and TNC (P>0.05, n=3). Data are presented as whisker–box plots with all points (whisker: full range; line: median; cross: mean). Kruskal-Wallis test followed by post hoc Dunn’s test was used for statistical analysis. Left panel shows nuclear DAPI staining. Middle panel shows green fluorescence-labeled COX-2, and right panel shows merge images. Scale bar indicates 50 μm (B) and 20 μm (C), respectively. [file 10194_2022_1384_MOESM2_ESM.jpg]
